# Convergence insufficiency and accommodative insufficiency in children

**DOI:** 10.1186/s12886-019-1061-x

**Published:** 2019-02-21

**Authors:** Amélia F. Nunes, Pedro M. L. Monteiro, Francisco B. P. Ferreira, António S. Nunes

**Affiliations:** 1 Department of Physics, University of Beira Interior, 6200 Covilhã, Portugal; 2 Departamento de Ciências Sociais e Humanas, Universidade da Beira Interior, Covilhã, Portugal; 3 Centro de Investigação em Ciências da Saúde (CICS), Covilhã, Portugal; 4 Centro Clínico e Experimental em Ciências da Visão, Covilhã, Portugal; 5 ubimedical, Covilhã, Portugal; 6 Núcleo de Estudos em Ciências Empresariais (NECE), Covilhã, Portugal

**Keywords:** Convergence insufficiency, Accommodative insufficiency, Normal binocular vision, Visual discomfort, Children

## Abstract

**Purpose:**

Convergence and accommodative insufficiency represent the main cause of complaints during close visual work and can reduce visual performance and comfort. Knowing their prevalence among schoolchildren is fundamental to define strategies for action. The purpose of this study was to estimate the frequency of these conditions in children in 5th and 6th school years in inland Portugal and to assess the impact that each visual condition has on their quality of life, based on the level of visual symptoms.

**Methods:**

a cross-sectional study was carried out with children enrolled in the 5th and 6th school years. 372 children (192 girls) were assessed, with average ages of 10.9 ± 0.9 years. Refractive error and binocular vision assessment, integrating accommodative parameters, were used to analyse the visual condition. Symptoms were quantified using the Portuguese version of the CISS (Convergence Insufficiency Symptom Survey).

**Results:**

The prevalence of definite Convergence Insufficiency (CI) in the children assessed was 2%. A prevalence of 6,8% could be considered if clinically significant CI (high suspect and definite categories) cases are accounted. In relation to Accommodative Insufficiency (AI), a frequency rate of 10% was recorded, with 3% of the evaluated children presenting AI and CI simultaneously. The symptoms score was higher in AI than in CI.

**Conclusions:**

A frequency of approximately 10% was found for each one of the visual syndromes, and it was verified that visual discomfort is common among teenagers who carry these conditions. In cases of asthenopia, such as headaches and loss of concentration, associated with near vision activities, there is a requirement to evaluate the quality of binocular vision.

## Introduction

Convergence Insufficiency (CI) is a binocular vision dysfunction, characterised by the patient’s inability to accurately converge, or sustain accurate convergence when focussing on near objects. It is generally associated with symptoms such as eyestrain, blurry vision, double vision, headaches and reading related problems [1]. Accommodative insufficiency (AI) is a condition that affects the ability to maintain near vision focus for a prolonged time. AI has been reported to be a common cause of asthenopia and other symptoms, in schoolchildren, associated with near vision [2]. The inability to concentrate for long periods during near visual work can reduce the level of student achievement, so CI and AI are presented as negative factors in relation to health and quality of life, as both interfere with reading and near work, contributing to diminished performance at school [1, 3, 4]. The literature has shown a high rate of comorbidity of CI and AI [5].

Concerning the rates of CI in general populations, several studies show different results [1, 6]. Among the various studies published, the data related to the prevalence of this condition range from 1.7 to 33% [1, 7]. These discrepancies can be attributed to various factors, from different exclusion criteria (samples not representative of the general population) to measurement methods and diagnostic criteria [1, 6–8]. An average in the order of 5% has been taken as the rate of prevalence of CI in the general population [1, 7]. This rate varies tremendously with population characteristics (e.g. age) and with the CI definition used (1 or more criteria). Studies in children have higher rates, and the frequency is reduced with the increase in criteria for diagnosis of CI [6, 7, 9, 10].

Researchers from the CITT group (Convergence Insufficiency Treatment Trial Investigator Group) have quantified the symptoms reported by patients with a questionnaire developed for this purpose, the CISS (Convergence Insufficiency Symptom Survey). This questionnaire has been validated for various populations, for different age-groups [11–14] and has been used regularly to help with diagnosis and to assess the effectiveness in treating the syndrome [4–6].

As stated by the CITT group, the presence of one, or more than one sign of CI allows classification of the visual condition at different levels, from suspected to definite [8]. This procedure of classifying cases of CI has been followed by other authors [5, 9, 10], which has led to comparable results. In addition, due to the high rate of comorbidity of CI and AI, several authors have evaluated the frequency of AI in their studies [5, 9]. Usually AI was defined by reduced accommodative amplitude, high values on monocular estimation retinoscopy and failing monocular accommodative facility with minus lenses [6, 8, 15, 16].

Review studies carried out with school samples, have reported AI rates ranging from 0.2 to 32.5% [16, 17]. Some studies do not mention the rate of comorbidity of AI and CI but in those that do, a rate that varies between 1.9 to 14.7% for AI with CI is reported. The factors that may contribute to these differences are identical to those described in CI (different exclusion criteria, measurement methods, and diagnostic criteria across studies).

Despite the large number of studies in this field with schoolchildren, it is noted that most of them have been carried out in the United States of America (USA). Epidemiological studies in Europe, are less frequent, and this is a limiting aspect for the knowledge of their prevalence by age groups. The aims of this study have been to estimate the frequency of Normal Binocular Vision (NBV), CI and AI in children attending the 5th and 6th school years, in a city in inland Portugal, quantify the level of symptoms with a scientifically validated scale in each case and assess the relation between CI, AI and visual symptoms. This research is very important since, (1) it is the first in Portugal; (2) it reports on frequency of NBV, CI and AI in a school-based sample (rather than clinic based) and includes students with significant but corrected refractive error (therefore, the sample is more representative of a school population); and (3) it presents the impact that each visual condition has on the quality of life, based on the level of visual symptoms.

## Subjects and methods

This is a cross-sectional study focused on children attending the 5th and 6th school years. It was authorised by the General Direction of Education (process n° 0410800001) and by the Commission for Ethics of the Faculty of Health Sciences at Beira Interior University (process CE-FCS-2012-027).

### Subjects

All children in the 5th and 6th years from the city’s schools were invited to participate in the study. The evaluated volunteers presented a free consent form, properly signed by their parents or legal guardians, authorising participation in the study.

### Procedures

The data collection was carried in the school over a three months. The room provided by the school was suitably adapted, ensuring adequate conditions in terms of test distances and lighting levels.

The protocol of visual assessment was adapted from the protocol of clinical procedures proposed by the CITT group, and as followed in others studies [5, 9, 16, 18]. Data on refraction, binocular vision and accommodative function was collected. All measurements were performed with their habitual refractive prescription.

#### Refractive measures

- Measurement of habitual refractive prescription with a lens meter.
- Visual Acuity (VA) at distance with ETDRS (Early Treatment of Diabetic Retinopathy Study) charts: monocular measurements letter by letter; VA at near with ETDRS charts, (right eye measurement only).
- Retinoscopy over the habitual refractive prescription.
  If children were found with a poor VA (worse than 0.1 logMAR) or a significant uncorrected refractive error (greater than 0.50 myopia, 1.00 hyperopia or astigmatism) they were referred to an optometric clinic setting, due to suspected uncorrected refractive error.

#### Binocular vision measures

- Distance and near phoria with cover test, prism bar and a Sloan letter (20/30 size at 4 m or 40 cm) as stimulus. The prism neutralization endpoint was recorded as the highest prism power that induced no movement before reversal of the deviation. If a neutral endpoint was not demonstrated using the prism amounts available in the prism bar, the endpoint was defined by averaging the largest prism power demonstrating the initial directional movement and the smallest prism power to cause reversal of movement [19].
- Negative and Positive Fusional Vergences at near with horizontal prism bar and a column of Sloan letters 20/30 size at 40 cm as stimulus. The test was stopped at the point of consistent diplopia.
- Near Point of Convergence with RAF (Royal Air Force) rule and as stimulus a column of letters 20/30 size at 40 cm. The test was stopped at the point of consistent diplopia. Recorded to the nearest half-centimetre (average of 3 measures).
- Stereopsis with Random dot test 500”arc. Used only to search for the presence of stereoscopic acuity.

#### Accommodation

- Amplitude of accommodation with RAF rule (push up and push down) and a line of letters 20/30 size at 40 cm as stimulus. Recorded to the nearest half-centimetre, only for the right eye, according to clinical protocols suggested in others studies [9, 11, 20]. For the push-up method, the subjects initially viewed the target at a distance of approximately 40 cm and then the target was moved slowly toward the child along the ruler. The test was stopped at the point of consistent blur, not the first blur. In the push-down method, the accommodative target was advanced toward the subject until a significant blur was produced, and then the target was pushed away until the subject could just read the letters [21]. For the analysis, the shortest measurement between the two methods was used.
- Monocular accommodative facility with Flipper ±2.00 and the Minnesota Low Vision Reading (NMREAD) chart (0.2 logMAR) as stimulus; right eye measurement only.

**Fig. 1:**
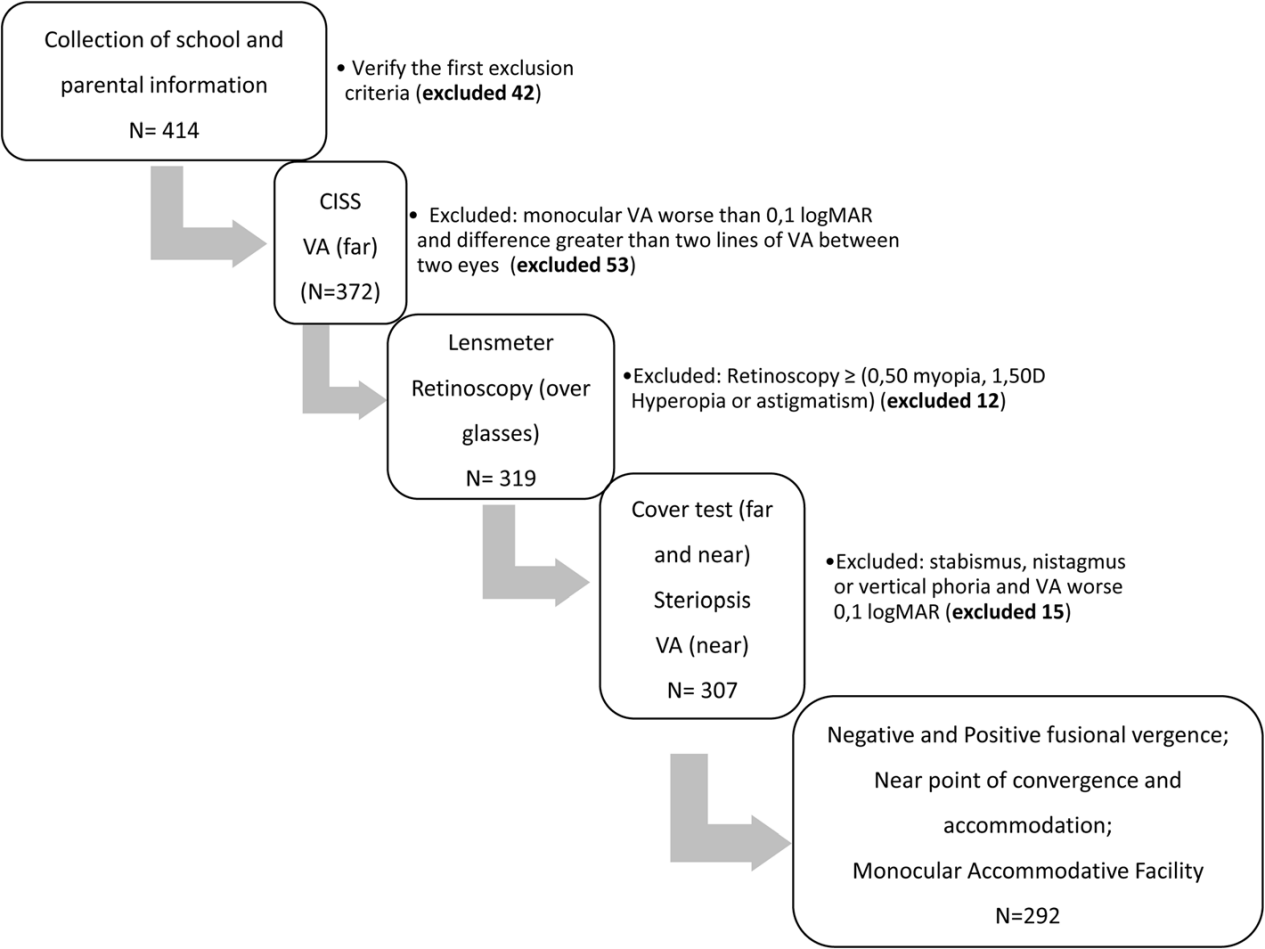
Scheme of examination procedures. (CISS- Convergence Insufficiency Symptom Survey; VA – Visual Acuity)

Data was acquired in the following order: Symptoms (CISS questionnaire Portuguese version); Refractive measures; Binocular Vision and Accommodative measures according to the scheme shown in Fig. 1.

### Exclusion criteria

Children were excluded if they had learning disabilities (e.g. attention deficit hyperactivity disorder), developmental delay or ocular surgery, reported by parents or inferred by poor cooperation. In addition, children were excluded if they had changed glasses in the last two weeks or present constant strabismus, nystagmus or vertical phoria at distance or near.

Data was also excluded from children who presented visual Acuity at distance or near, worse than 0.1 logMAR (0.8 in decimal system) in at least one of their eyes, or a difference greater than two lines of VA between the two eyes; with uncorrected refractive errors greater than 0.50D in Myopia, 1.50D in Hyperopia and Astigmatism.

### Criteria for CI, AI and NBV classification

For CI classification, the guidelines proposed in more recent studies about CI were followed, where the condition is classified in cases of suspected and cases of definite CI [7, 9, 10]. Suspected CI has been subdivided in cases of low or high suspect, according to the number of signs presented. For AI the same criteria as other similar studies, were used [5, 15].

To classify students with NBV, all the signs described in Table 1 (from 1 to 7) had to be verified. To classify CI, a low suspect CI presented the 8th criterion; a high suspect CI presented criterion 8 and other criterion (9 or 10); a definitive CI presented three criteria (from 8 to 10). To classify AI, the two signs described in Table 1 had to be verified. The cases that did not fit the previous ones were classified as Other Binocular Dysfunctions (OBD).

**Table 1:** Signs characterising normal binocular vision (NBV), convergence insufficiency (CI) and accommodative insufficiency (AI). (NFV-negative fusional vergence; PFV-positive fusional vergence; NPC-near point of convergence; RE-right eye)

Condition	Signs
NBV	1. Distance phoria: between 3∆ exo and 2∆ eso
	2. Near phoria: between 6∆ exo and 2∆ eso
	3. Near NFV ≥ x/7/3 (∆)
	4. Near PFV ≥ x/15/10 (∆)
	5. NPC break ≤6 cm
	6. Amplitude of Accommodation (RE): AA > (15–0,25*Age)-2 (Hofstetter’s minimum age formula)
	7. Monocular Accommodative Facility (RE): MAF ≥ 6 cpm
CI	8. Near exophoria 4∆ greater than distance phoria
	9. PFV break < 15∆ or failing Sheard’s criterion
	10. NPC break > 6 cm
AI	11. Monocular AA 2D ≤ Hofstetter’s minimum age formula: 15–0,25*Age
	12. MAF < 6 cpm (difficulty clearing − 2,00D)
OBD	OBD’s include children with tests outside normal limits not included in previously categories.

### Data analysis

Symptoms and age were summarized by mean and standard deviation. The statistical analysis to study the differences between groups was based on nonparametric tests, due to the great disproportionality between groups. The Mann-Witney test was used when studying the differences between two groups and the Kruskal-Wallis test when comparing the differences between more than two groups.

## Results

For this study, all students enrolled in the 5th and 6th year of education in the group of schools of the city were invited to attend. 372 children were assessed from a total of 412 students (response rate of 91%). Eighty were excluded because they did not meet the inclusion criteria. The assessment was completed by 292 children, 150 females and 142 males; 156 attended the 5th year, 137 attended the 6th. Participants’ ages ranged from 10 to 14 years old with a mean and standard deviation of 10.9 ± 0.8.

The eligible children (292), were grouped according to the classification criteria listed in Table 1, in four main categories: children with Normal Binocular Vision (NBV), children with Convergence Insufficiency (CI), children with Accommodative Insufficiency (AI), and children with other accommodative and nonstrabismic binocular dysfunctions (OBD).

The demographic characteristics for all volunteers assessed and for each clinical grouping are expressed in Table 2 (school year, gender and score symptoms reported by CISS questionnaire).

**Table 2:** Sample dimension and means and standard deviations of scoring on the CISS questionnaire. (N-sample size; CISS-convergence insufficiency symptom survey; SD-standard deviation; NBV-normal binocular vision; OBD-other binocular dysfunctions; CI-convergence insufficiency; AI-accommodative insufficiency)

Visual condition	N / %	School year (N)		Gender (N)		CISS
		5th year	6th year	Male	Female	Mean ± SD
NBV	165 / 56,5	86	79	86	79	7,99 ± 6,0
CI	41/14	23	18	21	20	11,55 ± 9,4
Low	21 / 7,2	10	11	9	12	9,71 ± 8,2
High	14 / 4,8	10	4	9	5	11,5 ± 9,3
Definite	6 / 2	3	3	3	3	19,17 ± 11,2
AI	29/10	19	10	12	17	18 ± 9,2
With CI	9 / 3,1	8	1	6	3	17,44 ± 7,8
Without CI	20 / 6,8	11	9	6	14	18,45 ± 10,0
OBD	66 / 22,6	47	40	36	51	12,49 ± 9,6

Considering that of all the children assessed 41 revealed the presence of at least one criterion for diagnosing CI, it is estimated that 14% of children attending the 2nd cycle of elementary education have CI, but only 14.6% (6/41) of these present a diagnosis of the dysfunction in its definite form. We found a rate of 6.8% CI in high suspect and definite forms.

It should also be noted that 29 students presented signs of AI (~ 10%) and 9 of these cases presented signs of CI with AI (one of low suspect CI, five of high suspect CI and three of definite CI).

Regarding the symptoms, there were no significant differences in the CISS score between gender or between school year.

The existence of an association between presence of the syndrome (CI) and the level of symptoms reported by the volunteers, based on the scoring of the symptoms on the CISS questionnaire, was evaluated (Pearson correlation: *N* = 206; R = 0.273; *p* < 0.001). In order to determine whether the level of symptoms reported by children with NBV and CI are significantly different, the Mann-Whitney test was used, revealing that there is statistical evidence to state that subjects with NBV have different symptom scores from subjects with CI (N = 206; U = 4.071; *p* = 0.044).

**Fig. 2:**
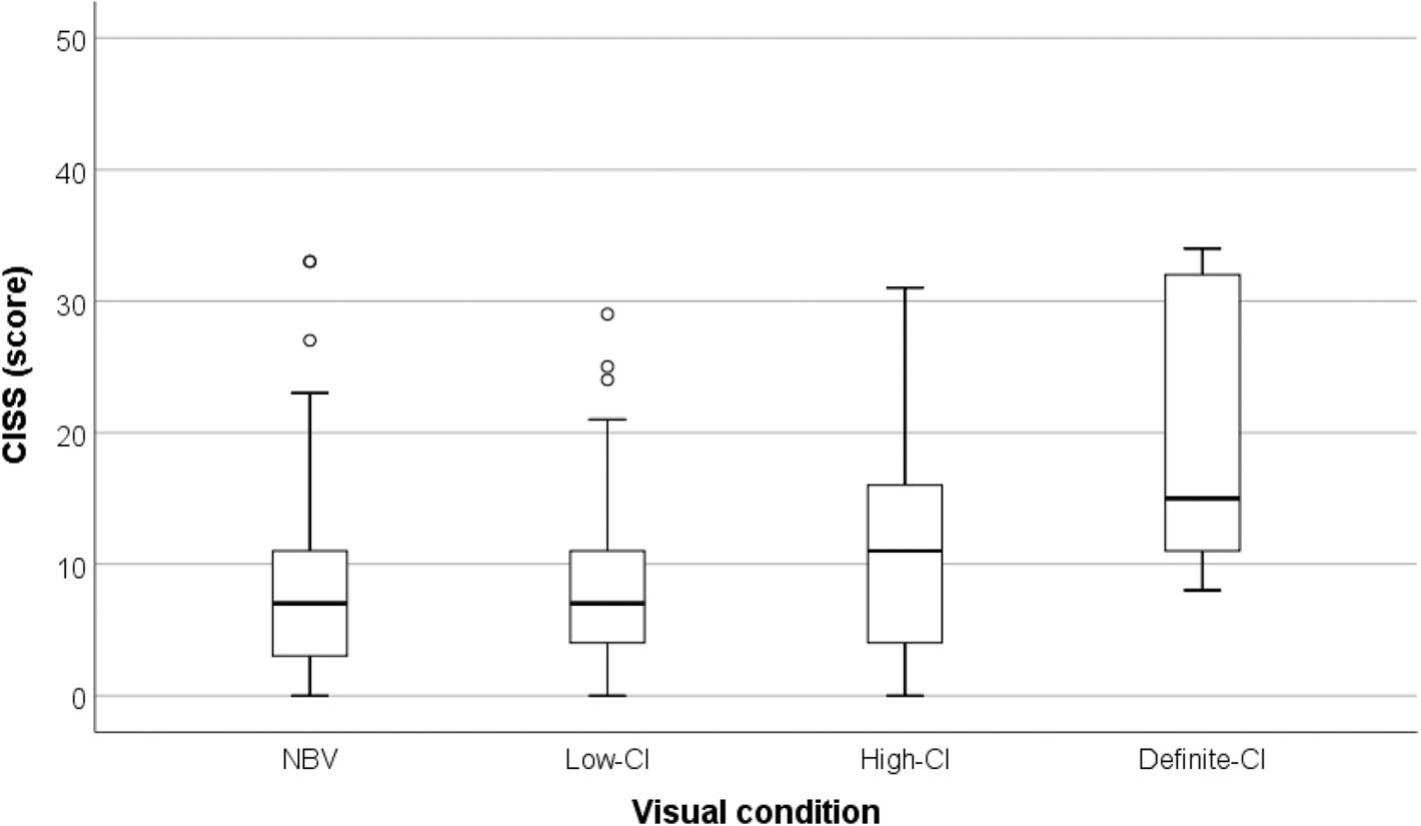
Distribution of scores on the CISS questionnaire (0–60), by different groups for CI levels. Low, high and definite CI refers to the number of CI signs, 1, 2 or more, respectively. An increase on the number of CI signs results in an increased CISS score

Grouping of CI into the three categories shows that a large percentage of cases found with this condition were classified as low suspect CI (51.2%), a condition requiring the lowest number of characteristic signs of CI. It is noted that the group of children with low CI is the one presenting a mean score on the CISS questionnaire closest to the mean score of subjects with NBV, as can be seen in Fig. 2. It can also be mentioned that the scores on the CISS questionnaire between students with NBV and students with low suspect CI are similar and the conditions of high suspect and definite CI are the ones presenting the highest scores on the symptom questionnaire.

To find out whether the different scores on the symptoms questionnaire between the different groups are significant, the differences were studied using the Kruskal-Wallis test, which showed significant differences in at least one of the categories (*N* = 206; H = 9.052; *p* = 0.029). Post-hoc analyses were carried out to identify which categories differ, regarding the level of symptomatology, and the results show that there is statistical difference only between NBV and CI in definitive form (*p* = 0.032).

**Fig. 3:**
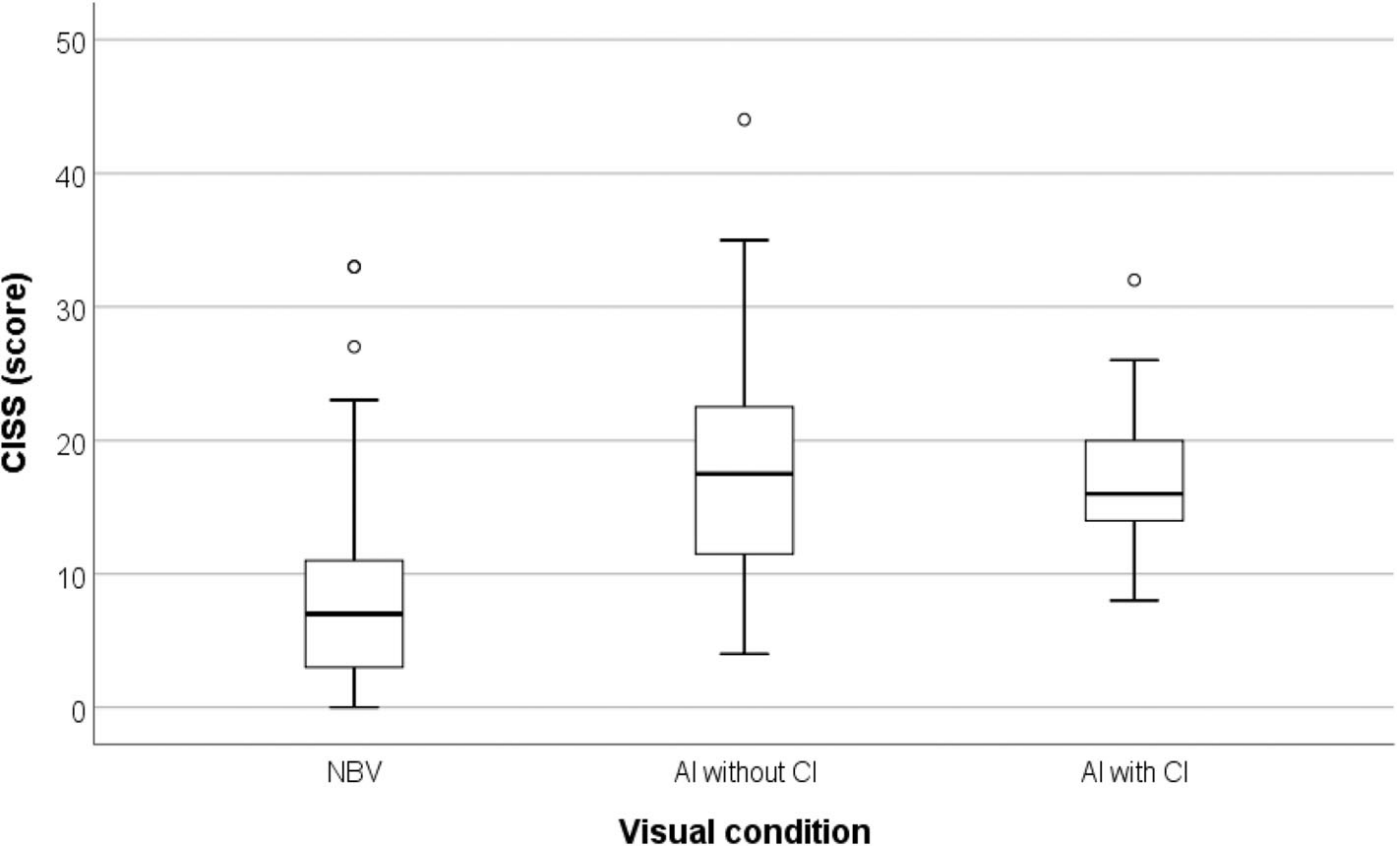
Distribution of scores on the CISS questionnaire (0–60), by AI (Accommodative Insufficiency), with and without CI. AI CISS scores are higher than NBV, regardless of CI presence

Comparing the level of symptoms among children presenting AI (with and without CI) and children presenting NBV (Fig. 3), it is observed that AI is more symptomatic and showed significant differences in at least one of the categories (Kruskal-Wallis test: *N* = 194; H = 3.397; *p* < 0.001). Post-hoc analysis shows that there are statistical differences between NBV and AI without CI (p < 0.001) and AI with CI (*p* = 0.001). Regarding CI with and without AI, statistical evidence was found to state that the differences are significant (Kruskal-Wallis test: *N* = 41; H = 6.524; *p* = 0.011)

## Discussion

This study revealed a frequency rate of CI in children of the 5th and 6th year of school that varied between 2% for a more conservative diagnosis, requiring the presence of the three criteria, to 14% for a more liberal diagnosis, requiring the presence of only one criterion. An intermediate prevalence of 6,8% can be considered if clinically significant CI (high suspect and definite categories) cases are accounted. The criteria applied for determining prevalence can change between authors and specific areas (Ophthalmology, Orthoptics, Optometry). In relation to AI, a frequency rate of 10% was recorded, and 3% of the evaluated children presented AI and CI together.

The CI rate found in this study is somewhat lower than the results reported in other studies with similar methodology (see Table 3). For the most significant forms of CI (high suspect and definitive), the rate of occurrence was 6.8%, while in other studies rates ranging from 7.75 to 20% were found [5, 9, 10, 18, 22]. Our results are closer to the results obtained by White and Wajuihian, which are related to prevalence studies in different geographic areas and countries with different degrees of development [10, 22].

**Table 3:** Rate of Convergence Insufficiency and Accommodative Insufficiency for age range between 7 and 19. (CI-convergence insufficiency; AI-accommodative insufficiency)

Study (Author/year)	Country	Age range	Sample size	Prevalence CI (%)			Prevalence AI (%)	
				Low	High	Definite	with CI	without CI
Rouse et al./1998 [7]	USA	8–12	428	33	12	6	–	–
Rouse et al./1999 [9]	USA	9–13	453	8,4	8,8	4,2	9,9	11,5
Borsting et al. /2003 [2]	USA	8–15	392	10,5	12,7	4,6	–	10,5
White and Major /2004 [22]	USA	7–19	129	–	7,75		–	–
Marran 2006 [5]	USA	11,5 ± 0,63	299	–	18		3,3	4,7
Davis et al./2016 [16]	USA	8–16	484	–	31,4		14,7	17,8
Menjivar et al./2018 [18]	USA	9–14	282	–	19,8		8,2	**–**
Wajuihian and Hansraj/2016 [10]	S/Africa	13–19	1211	11,8	6	4,3	1,9	–
Hussaindeen et al./2016 [17]	India	7–17	920	–	16,5		0,2	
Present study	Portugal	10–14	292	7,2	4,8	2	3,1	6,8

Regarding the frequency of AI, a rate of comorbidity with CI not as high as that suggested by Rouse, nor as low as that proposed by Wajuihian was found, with values similar to those found by Marran [5, 9, 10]. CI prevalence studies are more frequent than AI, and thus the need for more AI research is stressed, as proposed [5].

For many authors, the presence of symptoms is also a condition to be taken into account for the diagnosis of CI. The data obtained in this study shows that the CISS symptom score in the NBV and the CI presents statistically significant differences, but CISS symptom score, in cases of suspected CI, shows levels similar to the NBV cases. It has also been shown that the CISS symptom score increases as the number of criteria for the diagnosis of CI increases. Other authors also concluded that the CISS correlates with the number of signs of CI [7], and the weak correlation between the evaluation of symptoms with the CISS and the CI diagnosis has also been mentioned [5, 18, 23]. Regarding AI symptoms, there were significant differences between the CISS symptom score in NBV and AI (with and without CI). These findings are in agreement with the conclusions of other authors that AI is more symptomatic than CI [5, 24].

In a first approach, it is expected that the prevalence of these conditions in Europe will be similar to that reported in the USA, but our results reveal lower rates for both CI and AI. These findings need to be clarified emphasizing not only aspects related to geographic location and socioeconomic issues, but also factors related to the design of epidemiological studies and clinical procedures.

Regarding the geographical location of the different scientific studies available, most of the reports on this subject, conducted in children and teenagers, focus on the USA [5, 9, 16, 18]. They are less frequent in others geographic regions [10, 17]. On the other hand, previous epidemiological studies on binocular alterations, carried out in Europe, were performed with university students or generalised age groups [23, 25, 26], which makes it difficult to compare the results obtained in children or adolescents, as they differ of how they spend their time (from leisure time to food habits). The level of development of a country has an impact on its educational level and access to new technologies, which are factors that affect the lifestyles of its populations, and consequently visual effort with near activities. CI has been associated with the visual demand for school requirements, so it is expected that in rural areas and in less developed countries there will be a lower rate of CI than in more developed countries and urban areas. However, this aspect has not been advanced in scientific research. One recent study seeks to distinguish these two geographical areas and although the rural area has revealed a lower rate, it is in the same order of magnitude of the rate found in urban areas [17]. In our study, all children were evaluated, and the sample consisted of children from various social strata.

Although many of the procedures are comparable across studies, there are always some methodological differences. It was verified that the measurement of phoria in some studies, with age groups close to the present one, was performed using the Von Graefe technique [5, 9, 10], in others with the Thorington method [18]. In this study, we used the objectively neutralized cover test. Comparative studies of phorias measurement between the cover test and other techniques, conclude that the measure obtained by the cover test leads to lower magnitudes [27, 28]. However, the methodology applied in this study was the most suitable for the type of work performed, since it required the use of portable material to permit the evaluation of all the children in their normal school environment.

Another aspect that also deserves attention is related to the exclusion criteria. Very specific criteria, tend to reduce the sample size and skew epidemiological data. In some studies, the reported prevalence rates include only students with small refractive errors [5, 9], and/or are performed in clinical settings, and this might result in a bias, since the most deprived children tend not to participate in these studies. In this study, only children who did not cooperate in the evaluation and children with significant uncorrected refractive errors were excluded, so it is believed that the estimated rate is closer to the true values.

It is important to highlight the greater intensity of symptoms in children with these syndromes, especially those with an accommodative insufficiency. Other authors have also reported this relationship, noting that accommodative changes are more symptomatic than changes in convergence [5]. In addition, studies that have investigated the relationship between the intensity of near-work and visual complaints, found an association between the cumulative amount of near work, decreased accommodative facility and increased asthenopia [29].

## Conclusion

It is estimated from this study that the frequency of CI in Portuguese children aged between 10 and 14 years old, is slightly lower than the frequency of the same ages reported in other developed countries. The frequency of clinically significant CI (two or more signs) was only 6.8%. Comorbidity with AI was also identified and with rates similar to those reported in the literature. However, an AI rate (with and without CI) was slightly higher than the rate of significant CI. On the other hand, it was verified that AI is a visual condition with similar frequency to CI, which requires additional investigation. The visual complaints associated to these conditions suggest a decreased visual efficiency that can result in a poor school performance. The complaints of visual discomfort are broad and in clinical examination may not be associated with an increased effort on ocular convergence and focus. Kids and teenagers with headache complaints, somnolence while reading, loss of concentration in performing near vision activities, among other visual stress complaints, must be submitted to an evaluation of binocular vision and accommodation status.

## Abbreviations

AI: Accommodative insufficiency
CI: Convergence insufficiency
CISS: Convergence insufficiency symptom survey
CITT group: Convergence insufficiency treatment trial investigator group
ETDRS: Early treatment of diabetic retinopathy study
NBV: Normal binocular vision
NMREAD: Minnesota low vision reading
OBD: Other binocular dysfunctions
RAF: Royal air force
USA: United States of America
VA: Visual acuity
